# *luxRI *homologs are universally present in the genus *Aeromonas*

**DOI:** 10.1186/1471-2180-7-93

**Published:** 2007-10-23

**Authors:** Kamlesh Jangid, Richard Kong, Milind S Patole, Yogesh S Shouche

**Affiliations:** 1 Molecular Biology Unit, National Centre for Cell Science, University of Pune, Pune- 411007, Maharashtra, India; 2 527 Biological Sciences, Department of Microbiology, University of Georgia, Athens, GA-30602, USA; 3 Department of Biology and Chemistry, Centre for Coastal Pollution and Conservation, City University of Hong Kong, 83 Tat Chee Avenue, Kowloon Tong, Hong Kong Special Administrative Region, China

## Abstract

**Background:**

*Aeromonas *spp. have been regarded as "emerging pathogens". Aeromonads possess multifactorial virulence and the production of many of these virulence determinants is associated with high cell density, a phenomenon that might be regulated by quorum sensing. However, only two species of the genus are reported to possess the *luxRI *quorum sensing gene homologs. The purpose of this study was to investigate if the *luxRI *homologs are universally present in the *Aeromonas *strains collected from various culture collections, clinical laboratories and field studies.

**Results:**

Of all the 73 *Aeromonas *strains used in the study, seventy-one strains elicited acyl-homoserine lactone-mediated response in multiple biosensor strains. However, dot blot hybridization revealed that the *luxRI *homologs are present in all the strains. PCR amplification and sequencing revealed that the *luxRI *homologs shared a very high percentage sequence similarity. No evidence for lateral gene transfer of the *luxRI *homologs between aeromonads and other genera was noted.

**Conclusion:**

We propose that the *luxRI *quorum sensing gene homologs are universally present in the genus *Aeromonas *independently from their origin. This study is the first genus-wide report of the taxonomic distribution of the *luxRI *homologs.

## Background

The genus *Aeromonas *is a medically important genus in the family *Aeromonadaceae *within the *γ-Proteobacteria *[1]. *Aeromonas *species, referred to as "emerging pathogens" [2], are suspected to cause multiple infections in humans [3,4]. In addition, they cause diseases in amphibians, reptiles and fish [5]. They are more frequently isolated from samples of medical importance than from environmental sources [6,7]. Their multifactorial virulence determinants include surface associated factors like adhesins, extracellular proteins like siderophores for iron acquisition, and exoenzymes and exotoxins like α-haemolysin and serine proteases amongst others. The expression of many of these virulence determinants is associated with high cell densities [8-11] and are therefore putatively under control by quorum sensing.

Quorum sensing is a density-dependent regulation of the gene expression by self-generated signal molecules, such as the acyl-homoserine lactones (AHLs) in gram-negative bacteria. In *A. hydrophila*, the serine- and metalloprotease activities [12,13], biofilm development [14], and butanediol fermentation [15] are under quorum sensing control. Although, the AHL mediated production of extracellular proteases in *A. hydrophila *is decreased in the presence of long chain AHLs [12], mutations in its *luxRI *homologs do not affect its virulence towards gnotobiotically cultured *Artemia franciscana *[16]. Hence, a decrease in the expression of a virulence factor does not necessarily correlate with decreased virulence. Unfortunately only two species of this genus, *A. hydrophila *and *A. salmonicida*, are known to harbour the quorum sensing mechanism as opposed to the majority of *Aeromonas *spp. with known pathogenic potential [17]. It therefore necessitates investigating the distribution of this mechanism throughout this genus.

Despite the diversity of the phenotypes that are regulated by the quorum sensing network, *luxR *and *luxI *constitute evolutionary conserved gene families. *luxRI *homologs can be identified in most species in which AHL based quorum sensing is known to operate, although some alternative AHL synthases do exist [18]. Species can also possess differing number of *luxR *and *luxI *homologs or even a *luxR *homolog alone [19]. The AHL-mediated gene expression machinery is reportedly conserved within a particular genus and the species within that share very high sequence similarities with each other. In-between various genera within a family, even though there are certain highly conserved regions, the overall levels of sequence similarity are often very low and range between 18–25 % and 28–35 % for LuxR and LuxI homologs, respectively [20].

The environmental distribution of the AHL-mediated gene expression systems amongst bacteria is very poorly understood. Merely 2.2 % (21 bacterial genera) of the total number of bacterial genera listed in the Bergey's Manual of Systematic Bacteriology [21], are known to harbour the AHL producing species, and all of which belong to the *α-, β- *and *γ-Proteobacteria *only [22]. Unfortunately, at the species level this percentage drops to a fraction of a percent. Although few reports are known about the AHL-mediated gene expression by bacterial strains isolated from contact lens wearers [23], marine snow [24], and rumen [25], not many reports are available on the existence of this system across bacterial genera or within a genus. For instance, only two species of the genus *Aeromonas *are known to harbour the genes for quorum sensing mechanism [17]. The importance of this mechanism demands that the existence and distribution of AHL-mediated gene expression systems be studied across bacterial taxa.

With a view that there is a considerable sequence similarity in the AHL-mediated machinery within a genus, it may be possible to PCR amplify the *luxRI *homologs either individually or together. This hypothesis was tested in the present study using the genus *Aeromonas *as a model system to investigate if the *luxRI *quorum sensing gene homologs are universally present in this genus.

## Results

### AHL production by *Aeromonas *strains

**Figure 1:**
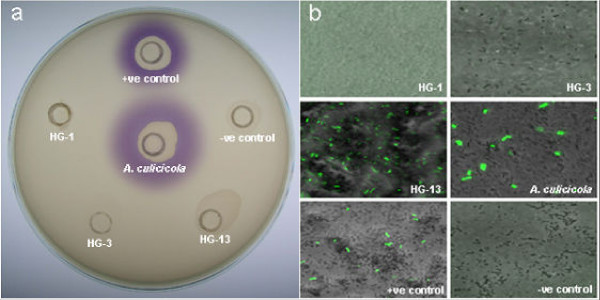
**Biosensor based detection of AHL production by *Aeromonas *strains**. a) *Chromobacterium violaceum *CV026 assay plate with both controls alongwith *A. culicicola *MTCC 3249^T ^and the three negative strains; and b) CLSM images of *gfp *expression from *E. coli *JM109 harboring pJBA89 in the presence of spent culture supernatants from all the six strains as in a) showing positive result for HG-13.

A total of 71 strains tested positive for AHL production. Of the 73 strains screened, 70 strains elicited AHL-mediated violet pigmentation in the *Chromobacterium violaceum *CV026 strain within 24 h. Amongst the three remaining strains which tested negative with the CV026 assay, one strain, *Aeromonas *sp. ATCC 43946^T ^(Hybridization Group, HG-13) elicited *gfp *expression in *E. coli *JM109 harboring pJBA89 in 24 h as observed in CLSM imaging analysis (Fig. 1). Two strains: *A. hydrophila *ATCC 7966^T ^(HG-1) and *A. hydrophila *CDC 0434-84 (HG-3) tested negative in all the three bioassays. No variation in the observations was noted when extracted AHLs from culture supernatant were screened similarly. Surprisingly, no inhibition of violet pigmentation in the CV026 reverse assay was noted for the three negative strains.

### Molecular detection of the *luxRI *homologs

**Figure 2:**
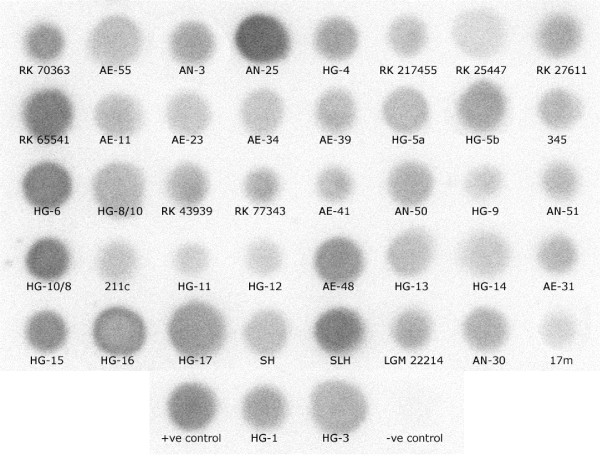
**Dot-blot analysis of *luxRI *gene homologs from *Aeromonas *strains**. A representative dot-blot showing the presence of *luxRI *homologs in all the strains that showed no PCR amplification for either *luxR *or *luxI *homolog. The negative control (genomic DNA of *Selenomonas lipolytica*) and positive control (genomic DNA of *A. culicicola*) are also shown.

All the seventy-three strains possessed the *luxRI *homologs as revealed by dot blot hybridization. Interestingly, the three strains that tested negative with the CV026 bioassay showed the presence of *luxRI *homologs (Fig. 2). Although, the PCR amplification of the complete regulon resulted in no success that of the individual gene fragments (*luxR *and *luxI *homologs) was successful. The amplification of the *luxR *homolog using the gene specific primer pair, QS-722F and QS-1444R produced a reproducible single DNA fragment (~790 bp) from seventy strains. The three exceptions were: *A. caviae *strains RK 217455 and RK 25447, and the recently reported *A. molluscorum *LMG 22214 ^T ^[26]. The amplification of the *luxI *homolog using the degenerate primer pair QS-24F and QS-697R resulted in partial success. PCR amplification with the primer pairs designed specifically for the *luxRI *homologs from *A. culicicola *MTCC 3249^T^, AcuRF/AcuRR and AcuIF/AcuIR, resulted in desired size fragment amplification for more strains. All these PCR products were then purified, sequenced and confirmed to be *luxRI *homologs. We were successful in PCR amplifying the *luxR *and *luxI *homologs from 70 and 34 strains, respectively.

### *luxRI *homolog sequence analysis

**Figure 3:**
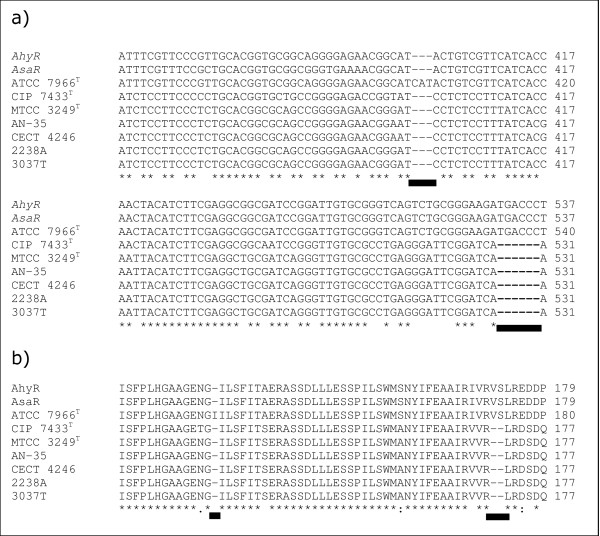
**Sequence alignment of the region with indels in the *luxR *homolog**. a) Nucleotide sequence; and b) corresponding amino acid sequence. Sites of insertion/deletion are marked by a solid line.

The *luxR *homologs showed a wide range of sequence similarity. The percentage sequence similarity ranged from 79.28–100 % corresponding to 0–161 nucleotide differences (Additional file 1 available online). Similarly, the inter-species nucleotide substitution rates were spread out (0.89–20.46 %). While the least values were shared between the type strains of *A. bestiarum/A. caviae *and *A. bestiarum/A. encheleia*, the maximum values were between *A. media *(HG-5A)/*A. culicicola*. Similar nucleotide substitution rates were also observed at the intra-species level (0.26–17.24 %). The *luxR *homologs varied in size from 777–786 nucleotides. A total of 331 variable positions (42.11 % of the sequenced region) of which a very high proportion of parsimoniously informative sites (*P*_*i*_) was observed (31.67 %). Indels were noted at specific positions in nine strains (Fig. 3). *A. hydrophila *ATCC 7966^T ^possessed a single triplet insertion (CAT) at position 402 while deletion of two nucleotide triplets was detected at position 530 of the *luxR *homologs in six other strains (*A. sobria *CIP 7433^T^; *A. culicicola *strains MTCC 3249^T^, SH and SLH; *A. veronii *bv sobria CECT 4246; and *A. trota *AN-35). The nucleotide positions are as per the *luxRI *homologs [GenBank:X89469] in *A. hydrophila *[17].

The *luxI *homologs showed lower sequence similarity. The percentage sequence similarity ranged from 69.34–100 % (Additional file 2 available online). The *luxI *homolgs varied in length from 624–639 nucleotides. A total of 237 *P*_*i *_sites (82 % of the total 289 variable positions) were observed. Similar to the *luxR *homologs, indels were noted in the *luxI *homologs from nine strains. However, three of these strains were different than the ones with indels in their *luxR *homologs. These strains were: *A. bestiarum *strains LMG 13448 and LMG 13662, and *A. veronii *bv sobria AE-21. In addition, these indels were detected in the 3' termini of the sequenced region with most lying in the primer-binding region (data not shown).

### Phylogenetic potential of *luxRI *homologs

**Figure 4:**
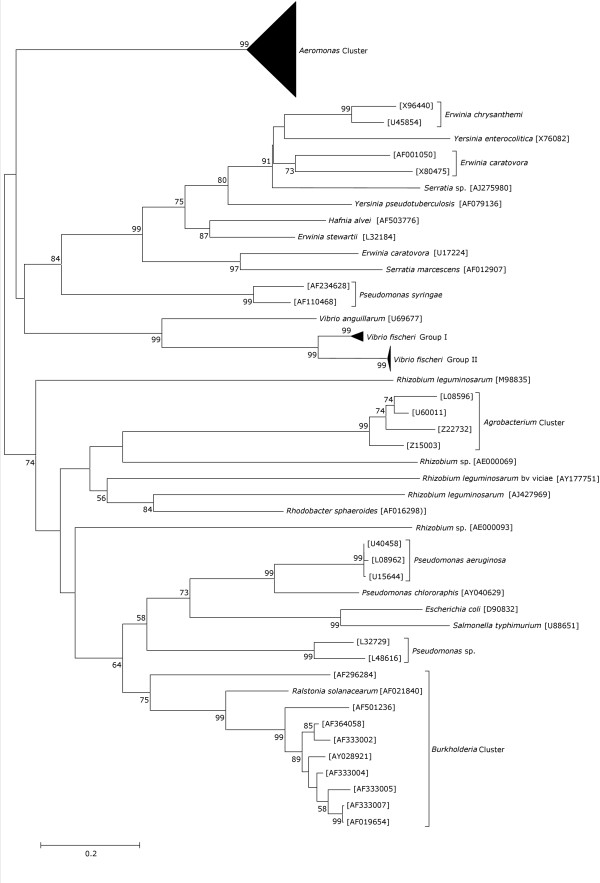
**Proteobacterial *luxR *homolog based phylogenetic tree**. The tree was constructed using the Kimura-2-parameter distances in the neighbor joining method. Values near the nodes represent percentage bootstrap support (1000 replicates). *Vibrio fischeri *Group I [GenBank:M96844, AY292966, AY292967, AY292969, AY292970, AY292979 and AY292980]; and *V. fischeri *Group II [GenBank:M19039, M25751, M25752, Y00509, AF170104, AY292964, AY292965, AY292971–AY292978, and AY292982–AY292985]. The GenBank accession numbers of the *luxRI *homologs from *Aeromonas *are given in Table 1.

The genus *Aeromonas *formed a distinct lineage, well separated from other genera in the class *Proteobacteria *for which the *luxR *(Fig. 4) and *luxI *homologs (data not shown) are known. However, some strains of other genera grouped with different genus, indicating possible events of lateral gene transfer (LGT) between them. Multiple tree topologies obtained using other tree-building algorithms were similar with some minor variations in the branch lengths. The phylogenetic trees were based on the nucleotide sequences rather than the amino-acid sequences due to the loss of information in the latter caused by synonymous substitutions as reported earlier [27]. The *luxI *homologs, similar to the *luxR *homologs, possessed the ability to discriminate the genus *Aeromonas *from the other genera in the proteobacterial class. However, the inability to sequence the *luxI *homologs from all the strains hampered further analysis for this gene.

## Discussion

The disruption of quorum sensing has been suggested as a new strategy for combating disease spread by pathogenic bacteria. Unfortunately, the scarcity of information on the environmental distribution of this mechanism and the fact that strains of the same species isolated from different ecological niches might possess diverse physiological characteristics, drastically limits the application of this strategy on a genus-wide basis. For instance, species of the genus *Aeromonas *have been isolated from diverse environments such as the midgut of *Culex quinquefasciatus *and *Aedes aegyptii *mosquitoes [28,29], monkey faeces [30], and bivalve molluscs [26], as well as from a variety of foods such as vegetables, raw milk, ice cream, meat and seafood [6,7]. The pathogenic potential of many species of genus *Aeromonas *indicates the presence of common regulatory machinery amongst strains isolated from different ecological niches. However, only two species of this genus are known to possess the quorum sensing mechanism [17]. In addition, only *A. hydrophila *has been studied in great detail [12-15]. An understanding of the genus-wide distribution of this mechanism in *Aeromonas *spp. is the first step towards ascertaining if there are certain common phenotypes under quorum sensing regulation in the genus *Aeromonas*. This will allow successful implementation of quorum sensing based disease control strategies.

Cell division in the genus *Aeromonas *may be linked to quorum sensing. Although, there is no experimental evidence, molecular studies in *A. hydrophila *and *A. salmonicida *[17] and our analysis of the quorum sensing network in *A. culicicola *MTCC 3249^T ^(K. Jangid, P. Verma, P. V. Parameswaran, M. S. Patole, and Y. S. Shouche, unpublished data) confirmed a gene with close homology to *iciA*, present downstream of the *luxRI *homologs. *iciA *is an *E. coli *gene encoding a specific inhibitor of chromosomal initiation of replication [31]. The presence of *iciA *in the genus *Aeromonas *clearly implicates that cell division may be linked to quorum sensing. The present study on the distribution of the quorum sensing regulatory network in the genus *Aeromonas*, therefore gains importance.

Earlier studies have reported that some bacterial strains may possess diverse signalling molecules, which cannot be detected by a single biosensor strain [32]. Using multiple biosensor strains would reduce such possibility. Although, possible AHL production from the majority of the strains was detected (Fig. 1), a conclusive evidence of the presence of *luxRI *homologs was obtained only after dot blot hybridization analysis (Fig. 2). We tested the extracted AHLs to confirm possible inhibition of biosensor strains by some secondary metabolites in the culture supernatant of the three negative strains. In addition, AHLs from upto 100 ml of culture supernatant of these three negative strains were also tested to determine if insufficient AHL quantities yielded the negative results. However, no variation in the results was noted. We hypothesized that regardless of the extent of *luxRI *homolog sequence similarity shared between *Aeromonas *strains, the AHLs produced may be diverse and thus not detected by the biosensor strains used. Gene sequencing analyses further validated this hypothesis. The three strains that tested negative in the CV026 bioassay or the reverse assay shared very high *luxR *homolog sequence similarity (94.58 % to 99.36 %) with the *ahyR *and *asaR *sequences [17] (Additional file 1 available online). Moreover, indels were noted in a group of strains at two localized regions in the autoinducer binding domain (amino acid 18–168) of the *luxR *homologs (Fig. 3). This domain specifically binds to the autoinducer molecules and these indels might therefore be important in autoinducer specificity.

The inability to PCR amplify the complete regulon and the *luxI *homologs from all strains confirmed previous observations that *Aeromonas *strains possess greater sequence diversity in *luxI *as compared to *luxR *[17]. Preliminary analysis of the percentage sequence similarity and the nucleotide substitution rates for the *luxRI *homologs support this observation. Our ongoing analysis of the quorum sensing network in *A. culicicola *MTCC 3249^T ^further supports the hypothesis (Jangid et al., unpublished data). We noted the presence of indels in the binding site of the designed degenerate primers for the *luxI *homolog from this strain and a 10 % lower sequence similarity value as compared to others [17]. This is contrary to the fact that the *luxI *homolog sequence similarities between genera are higher than the corresponding *luxR *homologs [20].

No evidence of LGT of the *luxRI *homologs to the genus *Aeromonas *from another donor was found. All the *Aeromonas *strains grouped together in a single cluster that was clearly separated from the other members within the *Proteobacteria *(Fig. 4). However, it is incorrect to conclude that it probably did not occur. It might be possible that if LGT occurred, the donor was probably an organism similar to *Aeromonas *but not so far included in the analysis. It is even possible that the lineage is currently extinct. From the depth of the branch (Fig. 4), it would seem likely that this organism would moderately be related to *Aeromonas *but certainly more related than any of the current genera used for the analysis. In case, there was no LGT and if paralogy occurred, some aeromonads or their ancestors might have had more than one gene for these proteins or that the properties of the proteins might be different for different aeromonads. Both of these scenarios have been discussed in great deal earlier [33]. The same study also reported that the overall congruity between the quorum sensing genes and the rRNA trees is consistent with an ancient origin for the quorum sensing proteins within the *Proteobacteria*. Our results were however, incongruent with these observations (data not shown). The potential of the *luxRI *homologs as molecular chronometer and its comparison with the conventional molecular chronometers for the genus *Aeromonas *has been studied in great detail (K. Jangid, J. M. Gonzalez, W. B. Whitman, G. B. Nair, R. Kong, M. S. Patole, and Y. S. Shouche, unpublished data).

## Conclusion

In conclusion, using both conventional bioassay and molecular approaches, we proved that all the *Aeromonas *strains used in the study possess the quorum sensing genes irrespective of the nature of the isolates, whether clinical or environmental. The universal presence of the *luxRI *homologs with high sequence similarity in this genus makes it a potential target for treating *Aeromonas *borne infections. An understanding of the signaling mechanism in *Aeromonas *strains from a particular ecological niche would provide a model for bacterial communication among such strains. To our knowledge, this is the first genus-wide study of the taxonomic distribution of *luxRI *homologs and is the first step in assessing the significance of this mechanism both as a survival strategy and in maintaining ecosystem function.

## Methods

### Bacterial strains, media, and culture conditions

A total of 73 *Aeromonas *strains collected from clinical as well as environmental sources and different geographical regions were used in the study (Table 1). Most of the clinical strains were isolated from patients with acute diarrhea over a period of 2 years in Kolkata, India [34]. Eight clinical strains were isolated from stools of diarrhea patients at Queen Mary hospital in Hong Kong, China. The eleven environmental strains were isolated from ovary, salivary gland, or midgut of wild mosquito species collected from across India over a period of 2 years. All these strains were identified based on conventional microbiological and biochemical analysis. In addition, two recently reported *A. culicicola *strains (2238A and 3037T) from a drinking water supply in Spain were also included [35]. The AHL responsive biosensor strains used were: *Chromobacterium violaceum *CV026 [36], *E. coli *JM109 harboring plasmid pSB403 [37] and *E. coli *JM109 harboring plasmid pJBA89 [38]. These three strains were selected based on earlier reports [36-39] that they could detect a wide range of AHL compounds (Table 2). Although, the plasmid pJBA89 [38] was originally derived from pSB403 [37], the ribosomal binding sites in the former was optimised to detect low concentrations of AHLs and has a *gfp*-fusion which provides low background fluorescence [for details see [38]]. After arrival and/or collection, each bacterial strain was checked for purity on solid medium, and its identity was confirmed by partial sequencing of the 16S rRNA gene. All the strains used in the study were maintained on Luria-Bertani (LB) medium at 30°C, except for the *A. salmonicida *strains, which were incubated at 25°C. Wherever necessary, the medium was supplemented with 50 μg of ampicillin ml^-1^, 40 μg of kanamycin ml^-1 ^or 20 μg of tetracycline ml^-1^. Cell growth was monitored by measuring the optical density at 600 nm.

**Table 1:** List of *Aeromonas *strains used in the study

**Species (Hybridization Group)**	**Strain No.**	**Isolation site**	**GenBank Accession No.**	
			
			**LuxI**	**LuxR**
***A. hydrophila *(HG-1)**	ATCC 7966^T^	Tin of milk with fishy odour	AY987564	AY764300
	ATCC 49140	Human	AY987586	AY764351
	RK 217215	Human faeces	AY987590	AY764358
	RK 70363	Human faeces	NS	AY764359
	AE-53	Patient with acute diarrhea	AY987572	AY764329
	AE-55	Patient with acute diarrhea	NS	AY764330
	AE-57	Patient with acute diarrhea	AY987573	AY764331
	AN-1	Patient with acute diarrhea	AY987574	AY764332
	AN-2	Patient with acute diarrhea	AY987575	AY764333
	AN-3	Patient with acute diarrhea	NS	AY764334
	AN-25	Patient with acute diarrhea	NS	AY764336
	AN-32	Patient with acute diarrhea	AY987577	AY764338
***A. bestiarum *(HG-2)**	ATCC 51108^T^	Infected fish	AY987565	AY764301
	ATCC 13444	Ditch water	AY987587	AY764352
	ATCC 23211	Water supply	AY987588	AY764353
	ATCC 23213	River water	AY987589	AY764354
	LMG 13448	Human faeces	AY987581	AY764346
	LMG 13662	Faeces	AY987582	AY764347
***A. salmonicida *(HG-3)**	CECT 894^T^	Salmon, Salmo salar	AY987583	AY764348
	CDC 0434–84	Freshwater	AY987566	AY764302
***A. caviae *(HG-4)**	ATCC 15468^T^	Epizootic of young guinea pigs	NS	AY764303
	RK 217455	Human faeces	NS	NS
	RK 25447	Human faeces	NS	NS
	RK 27611	Human faeces	NS	AY764355
	RK 65541	Human faeces	NS	AY987549
	AE-11	Patient with acute diarrhea	NS	AY764321
	AE-23	Patient with acute diarrhea	NS	AY764323
	AE-34	Patient with acute diarrhea	NS	AY987548
	AE-39	Patient with acute diarrhea	NS	AY764325
***A. media *(HG-5A)**	CDC 0862–83	Infected fish	NS	AY764304
***A. media *(HG-5B)**	ATCC 33907^T^	Fish farm effluent	NS	AY764305
	345	NA	NS	AY764344
***A. eucrenophila *(HG-6)**	ATCC 23309^T^	Freshwater fish	NS	AY764306
***A. sobria *(HG-7)**	CIP 7433^T^	Fish	AY987567	AY764307
***A. veronii *bv sobria (HG-8/10)**	CDC 0437–84	Infected fish	NS	AY764308
	CECT 4246	Frog red-leg	AY987580	AY764345
	RK 43939	Human faeces	NS	AY764356
	RK 77343	Human faeces	NS	AY764357
	AE-21	Patient with acute diarrhea	AY987570	AY764322
	AE-41	Patient with acute diarrhea	NS	AY764326
	AN-50	Patient with acute diarrhea	NS	AY764341
***A. jandaei *(HG-9)**	ATCC 49568^T^	Faeces from patient with diarrhea	NS	AY764309
	AN-51	Patient with acute diarrhea	NS	AY764342
***A. veronii *bv veronii (HG-10/8)**	ATCC 35624^T^	Sputum of drowning victim	NS	AY764310
	211c	NA	NS	AY764343
***Aeromonas *sp. (HG-11)**	ATCC 35941	Ankle suture	NS	AY764311
***A. schubertii *(HG-12)**	ATCC 43700^T^	Forehead abscess	NS	AY764312
	AE-48	Patient with acute diarrhea	NS	AY764327
***Aeromonas *sp. (HG-13)**	ATCC 43946	Human leg wound	NS	AY764313
***A. trota *(HG-14)**	ATCC 49657^T^	Human faeces	NS	AY764314
	AE-31	Patient with acute diarrhea	NS	AY764324
	AN-35	Patient with acute diarrhea	AY987578	AY764339
***A. allosaccharophila *(HG-15)**	CECT 4199^T^	Diseased elvers	NS	AY764315
***A. encheleia *(HG-16)**	CECT 4342^T^	Healthy juvenile freshwater eel	NS	AY764316
***A. popoffii *(HG-17)**	LMG 17541^T^	Drinking water production plant	AY987568	AY764317
***A. culicicola***	MTCC 3249^T^	Mosquito midgut	AY987569	AY764318
	SH	Mosquito midgut	NS	AY764319
	SLH	Mosquito midgut	NS	AY764320
	2238A	Domestic water supply	AY987596	AY987550
	3037T	Domestic water supply	AY987597	AY987551
***A. molluscorum***	LMG 22214^T^	Wedge-shells (*Donax trunculus*)	NS	NS
***Aeromonas *sp.**	AE-51	Patient with acute diarrhea	AY987571	AY764328
	AN-24	Patient with acute diarrhea	AY987576	AY764335
	AN-30	Patient with acute diarrhea	NS	AY764337
	AN-46	Patient with acute diarrhea	AY987579	AY764340
	Manipal A1	NA	AY987584	AY764349
	ABJ	Gastric biopsy of gastritis patient	AY987585	AY764350
	1 m	Mosquito ovary	AY987591	AY764360
	12 m	Mosquito ovary	AY987592	AY764362
	13 m	Mosquito ovary	AY987593	AY764363
	15 m	Mosquito ovary	AY987594	AY764364
	17 m	Mosquito salivary gland	NS	AY764365
	19 m	Mosquito ovary	AY987595	AY764366

NA, Data not available; and NS, not sequenced due to inability to PCR amplify the gene under the conditions used.

**Table 2:** Range of AHLs detected by the three biosensor strains used in the study

**acyl-homoserine lactone (AHL)**	**CV026**	**pSB403**	**pJBA89**
N-Butanoyl-L-homoserine lacone (BHL)	▲	◦	▲ ▼ ▲
N-Butanoyl-L-homocysteine thiolacone (BHT)	▲	◦	◦
N-(-3-Oxobutanoyl)-L-homoserine lacone (OBHL)	▲ ▼ ▲	◦	◦
N-Benzoylacyl-l-homoserine lactone (BAHL)	▲	◦	◦
N-Hexanoyl-L-homoserine lacone (HHL)	▲	▲	▲
N-Hexanoyl-L-homocysteine thiolacone (HHT)	▲	◦	◦
N-(-3-Oxohexanoyl)-L-homoserine lacone (OHHL)	▲	▲	▲
N-(-3-Oxohexanoyl)-D-homoserine lacone ((D)OHHL)	▲ ▼ ▲	◦	◦
N-(-3-Oxohexanoyl)-L-homocysteine thiolacone (OHHT)	▲	◦	◦
N-Octanoyl-L-homoserine lacone (OHL)	▲	▲	▲ ▼ ▲
N-(3-Oxo-octanoyl)-l-homoserine lactone (OOHL)	▲	▲	◦
N-Decanoyl-l-homoserine lactone (DHL)	△	▲	◦
N-(3-Oxodecanoyl)-l-homoserine lactone (ODHL)	△	▲	◦
N-Dodecanoyl-l-homoserine lactone (dDHL)	△	▲ ▼ ▲	◦
N-(3-Oxododecanoyl)-l-homoserine lactone (OdDHL)	△	▲ ▼ ▲	◦
N-Tetradecanoyl-l-homoserine lactone (tDHL)	△	◦	◦
N-(3-Oxotetradecanoyl)-l-homoserine lactone (OtDHL)	△	◦	◦

"▲" indicates a suitable biosensor; "▲ ▼ ▲" indicates that the sensor will detect the AHLs at high concentration; "△" indicates that the sensor will antagonize AHL-mediated induction; and "◦" indicates data not available. The data presented here was previously reported [36–39].

### Screening for AHL production

For preliminary screening for AHL production by the *Aeromonas *strains, 20 μl of the culture supernatant from overnight growth was inoculated in wells created onto LB agar plates seeded with 1 ml (OD_600 _= 1) of CV026 culture. Visible violet pigmentation was checked after 24–48 h incubation at 30°C. The assay with the other two biosensor strains was carried out as described earlier [40]. Bioluminescence was measured after 30 min incubation. Confocal laser scanning microscope (CLSM) imaging analysis of gfp expression by *E. coli *JM109 harboring plasmid pJBA89 was done at 10× using an argon laser at 488 nm on a Zeiss LSM510 (Jena, Germany). Synthetic AHL standards: BHL (catalogue no. 09945; Fluka), HHL (catalogue no. 09926; Fluka), OHL (catalogue no. 10940; Fluka), DHL (catalogue no. 17248; Fluka) and dDHL (catalogue no. 17247; Fluka) served as controls. Strains, tested negative were processed for AHL extraction from 5 ml culture supernatant using dichloromethane (7: 3::supernatant: dichloromethane). A parallel extraction of the AHLs from *A. culicicola *MTCC 3249^T ^served as a control. The dried extract was reconstituted in 50 μl HPLC grade acetonitrile, a 10 μl aliquot spot dried onto paper discs (Whatman 3 M) and processed similarly as above. To detect long-chain AHLs which antagonize activating signals, the three negative strains were also tested using the CV026 reverse assay as described by Swift [39].

### Dot blot hybridization, PCR amplification and sequencing

Genomic DNA was prepared by standard phenol/chloroform/isoamyl alcohol extraction [41]. For dot blot hybridization, 1–2 μg of genomic DNA was denatured and spotted (2 μl aliquots) onto positively charged Nylon membrane (Hybond N^+^, Amersham Pharmacia Biotech UK Ltd.). Hybridization was performed at 50°C for 14 h. Unbound DNA was washed off with 2× SSC/0.1 % SDS for 20 min at room temperature followed by a second wash with 0.2× SSC/0.1% SDS for 15 min at 42°C for another 20 min. Intensifier screens (Imaging Screen-K, Biorad, USA) were scanned after 30 min on Molecular Imager FX (Biorad, USA). The *A. hydrophila ahyRI *fragment [17] was used as the heterologous probe. The probe was random labeled with [α-^32^P]dATP using the Megaprime DNA labeling system (Amersham Pharmacia Biotech UK Ltd.) as per the manufacturer's instructions. The *luxRI *homologs were PCR amplified under following conditions: initial denaturation at 95°C for 3 min, 35 cycles of denaturation at 95°C for 1 min, annealing at 55°C for 1 min and extension at 72°C for 1 min followed by elongation at 72°C for 10 min. The details of the primers used are given in Table 3. The PCR amplified products were purified by PEG/NaCl precipitation and sequenced using the PCR primers either on an ABI-310 or ABI-3730 automated DNA analyzer (Applied Biosystems).

**Table 3:** List of primers used in the study

Primer Name*	**Primer Sequence (5' to 3')**
QSH-24F	TTA TTC TGT GAC CAG TTC GCG CGC
QS-24F	TTA YTC KGT GAC CAG TTC SCK SGC
QS-697R	GGT CTT GTT TCA TAT GCT AGC CCC C
QS-722F	GGG GGC TAG CAT ATG AAA CAA GAC C
QS-1444R	TTA TTG CAT CAG CTT GGG GAA GTT G
AcuIF	ATG TTG GTT TTC AAA GGA AAA TTG
AcuIR	TTA TAT CTG GGC CGC TAA CTC ATG GGA
AcuRF	ATG AAA CAA GAG CAA CTG TTT GAG TAT
AcuRR	CTA TTG CAT CAG TTT AGG GAA GTT GGT

'*'- Numbers in the primer name indicate the 3' end binding site with respect to *A. hydrophila *sequence [GenBank:X89469].

### Phylogenetic analysis

Nucleotide and protein sequence analysis was performed with BLAST [42]. The sequences were aligned using the CLUSTALW v1.83 at the European Bioinformatics site [43]. The GenBank accession numbers for the sequences are given in Table 1. In addition, the two previously reported *Aeromonas luxRI *homolog sequences [GenBank:X89469 and U65741] were also used. The sequence similarity matrix was prepared using the DNAdist program in the PHYLIP package [44] using the Jukes Cantor corrections. The phylogenetic trees were constructed with the neighbor joining method using Kimura-2-parameter distances in MEGA v3.1 [45]. The resulting trees were compared with the parsimony method (100 bootstrap replicates) in the PHYLIP package and with the maximum-likelihood method using the fastDNAml program [46].

## Authors' contributions

KJ carried out the molecular genetic studies, sequence retrieval and analysis and drafted the manuscript. KJ, RK, MSP and YSS conceived of the study, and participated in its design and coordination. YSS was also involved in the analysis and interpretation of results and drafting the manuscript. All authors read and approved the final manuscript.

## Supplementary Material

Additional file 1 Similarity Matrix for the *luxR *homologs. Percentage sequence similarity (lower left) and number of nucleotide differences (top right). Click here for file

Additional file 2 Similarity Matrix for the *luxI *homologs. Percentage sequence similarity (lower left) and number of nucleotide differences (top right). Click here for file
