# Supplementary material for: luxRI homologs are universally present in the genus Aeromonas
Source: BMC Microbiol. 2007 Oct 23;7:93. doi: 10.1186/1471-2180-7-93 (PMC2180181; doi:10.1186/1471-2180-7-93)
Supplement: Additional file 1 — Similarity Matrix for the luxR homologs. Percentage sequence similarity (lower left) and number of nucleotide differences (top right). [file 1471-2180-7-93-S1.pdf]

Additional Table 1: Similarity Matrix for the *luxR* homologs. Percentage sequence similarity (lower left) and number of nucleotide differences (top right).

		1	2	3	4	5	6	7	8	9
1	***A. hydrophila*** **(X89469)**		35	5	32	42	33	105	41	132
2	***A. salmonicida*** **(U65741)**	95.49		38	24	11	29	107	54	137
3	***A. hydrophila*** **ATCC 7966^{T}**	99.36	95.14		31	43	32	102	40	133
4	***A. bestiarum*** **ATCC 51108^{T}**	95.88	96.90	96.04		23	7	105	41	136
5	***A. hydrophila*** **CDC 0434-84**	94.58	98.58	94.46	97.06		24	111	57	141
6	***A. caviae*** **ATCC 15468^{T}**	95.74	96.29	95.88	99.11	96.90		106	44	136
7	***A. media*** **ATCC 33907^{T}**	86.46	86.33	86.84	86.46	85.69	86.46		116	144
8	***A. eucrenophila*** **ATCC 23309^{T}**	94.71	93.11	94.84	94.71	92.65	94.38	85.19		139
9	***A. sobria*** **CIP 7433^{T}**	83.01	82.37	82.71	82.50	81.67	82.50	81.47	82.11	
10	***A. veronii*** **bt *sobria* CDC 0437-84**	95.41	99.36	95.02	97.06	98.97	96.65	86.46	93.23	82.11
11	***A. jandaei*** **ATCC 49568^{T}**	96.78	95.02	96.43	95.49	93.93	95.62	86.59	93.56	82.24
12	***A. veronii*** **bt *veronii* ATCC 35624^{T}**	96.78	97.69	96.43	98.34	97.06	97.69	86.46	94.38	82.46
13	***Aeromonas*** **sp. ATCC 35941^{T}**	94.12	95.49	93.69	96.43	94.84	95.74	86.84	92.65	82.37
14	***A. schubertii*** **ATCC 43700**	92.08	93.11	91.62	93.93	92.21	93.49	85.69	90.17	84.66
15	***Aeromonas*** **sp. ATCC 43946**	92.52	94.46	92.65	94.12	94.12	94.25	84.16	90.71	79.60
16	***A. trota*** **ATCC 49657^{T}**	96.04	95.02	95.62	95.74	94.25	95.41	86.46	93.69	81.80
17	***A. allosaccharophila*** **CECT 4199^{T}**	96.65	95.02	96.29	95.62	93.93	95.49	86.84	93.36	82.50
18	***A. encheleia*** **CECT 4342^{T}**	96.52	97.55	96.17	99.11	96.90	98.47	86.72	95.14	82.46
19	***A. popoffii*** **LMG 17541^{T}**	95.28	96.65	94.84	97.69	96.04	97.06	86.08	94.12	81.80
20	***A. culicicola*** **MTCC 3249^{T}**	83.37	82.71	83.14	82.37	82.11	82.11	81.08	82.50	93.05
21	***A. culicicola*** **SH**	96.17	97.19	95.74	98.97	96.52	98.58	86.46	94.71	82.24
22	***A. culicicola*** **SLH**	83.01	83.37	82.46	82.59	82.34	82.12	81.22	81.43	84.79
23	***A. caviae*** **AE-11**	97.19	94.84	96.78	95.41	93.81	95.49	86.72	93.69	82.58
24	***A. veronii*** **bt *sobria* AE-21**	97.19	94.84	96.78	95.41	93.81	95.49	86.72	93.69	82.58
25	***A. caviae*** **AE-23**	96.29	96.52	95.88	98.08	95.88	97.94	86.59	94.38	82.24
26	***A. trota*** **AE-31**	97.32	95.14	96.90	95.74	94.25	95.88	86.84	93.81	83.01
27	***A. caviae*** **AE-34**	86.46	85.43	86.46	85.43	84.65	85.19	93.69	84.04	81.21
28	***A. caviae*** **AE-39**	97.06	95.14	96.65	95.74	94.25	95.88	86.59	93.81	82.50
29	***A. veronii*** **bt *sobria* AE-41**	97.06	94.71	96.65	95.28	93.69	95.41	86.59	93.56	82.46
30	***A. schubertii*** **AE-48**	96.65	94.38	96.29	94.84	93.36	95.02	86.33	93.36	82.24
31	***Aeromonas*** **sp. AE-51**	97.32	95.14	96.90	95.74	94.25	95.88	86.84	93.81	83.01
32	***A. hydrophila*** **AE-53**	97.32	95.02	96.90	95.49	93.93	95.62	86.84	93.81	82.58
33	***A. hydrophila*** **AE-55**	96.65	94.38	96.29	94.84	93.36	95.02	86.33	93.23	82.11
34	***A. hydrophila*** **AE-57**	97.32	94.84	96.90	95.49	93.93	95.41	86.72	93.93	82.71
35	***A. hydrophila*** **AN-1**	97.19	94.84	96.78	95.41	93.81	95.28	86.46	93.81	82.58
36	***A. hydrophila*** **AN-2**	97.32	95.02	96.90	95.49	93.93	95.41	86.59	93.81	82.71
37	***A. hydrophila*** **AN-3**	97.32	95.02	96.90	95.49	93.93	95.41	86.59	93.81	82.58
38	***Aeromonas*** **sp. AN-24**	97.32	94.84	96.90	95.49	93.93	95.62	87.10	93.81	82.71
39	***A. hydrophila*** **AN-25**	97.19	94.71	96.78	95.41	93.81	95.49	86.84	93.69	82.58
40	***Aeromonas*** **sp. AN-30**	97.19	94.84	96.78	95.41	93.81	95.41	86.59	93.69	82.46
41	***A. hydrophila*** **AN-32**	97.06	94.71	96.65	95.28	93.69	95.28	86.46	93.56	82.46
42	***A. trota*** **AN-35**	83.27	82.50	82.71	81.67	81.67	81.54	80.38	82.24	92.40
43	***Aeromonas*** **sp. AN-46**	96.90	94.58	96.52	95.14	93.56	95.28	86.59	93.49	82.50
44	***A. veronii*** **bt *sobria* AN-50**	96.52	94.25	96.17	94.71	93.23	94.58	85.81	93.11	82.11
45	***A. jandaei*** **AN-51**	97.19	94.84	96.78	95.41	93.81	95.49	86.72	93.69	82.58
46	***A. veronii*** **211c**	96.04	97.32	95.62	98.58	96.65	98.21	86.84	94.58	82.50
47	***A. media*** **345**	86.59	85.81	86.46	85.69	85.19	85.44	95.14	84.42	80.50
48	***A. veronii*** **CECT 4246**	83.01	82.24	82.46	81.41	81.41	81.34	80.63	81.80	93.44
49	***A. bestiarum*** **LMG 13448**	96.43	97.69	96.04	99.23	97.06	98.58	86.59	95.02	82.58
50	***A. bestiarum*** **LMG 13662**	96.29	97.55	95.88	99.11	96.90	98.71	86.72	94.84	82.46
51	***A. salmonicida*** **CECT 894^{T}**	95.41	99.87	95.02	96.78	98.47	96.43	86.46	92.90	82.24
52	***Aeromonas*** **sp. Manipal A1**	97.19	94.71	96.78	95.41	93.81	95.49	87.10	93.69	82.58
53	***Aeromonas*** **sp. BJMC**	96.65	94.25	96.29	94.84	93.36	95.02	86.46	93.23	82.37
54	***A. hydrophila*** **ATCC 49140**	97.55	93.56	97.19	93.93	92.90	94.38	85.43	93.11	81.47
55	***A. bestiarum*** **ATCC 13444**	95.14	99.11	94.71	96.78	98.71	96.43	86.46	92.90	82.11
56	***A. bestiarum*** **ATCC 23211**	94.84	98.84	94.46	96.52	98.47	96.17	86.21	92.77	81.67
57	***A. bestiarum*** **ATCC 23213**	96.17	97.32	95.74	98.71	96.65	98.34	86.46	94.58	82.24
58	***A. caviae*** **RK 27611**	96.90	94.46	96.78	95.14	93.81	95.28	87.10	93.69	82.50
59	***A. caviae*** **RK 65541**	86.21	85.44	86.33	85.81	85.31	85.56	92.90	83.75	81.34
60	***A. veronii*** **bv *sobria* RK 43939**	82.34	82.59	82.38	82.12	81.56	81.43	80.65	81.35	84.30
61	***A. veronii*** **bv *sobria* RK 77343**	83.14	83.49	82.59	82.59	82.46	81.81	81.48	81.56	84.66
62	***A. hydrophila*** **RK 217215**	96.43	93.93	96.04	94.84	93.11	94.71	86.33	92.90	82.50
63	***A. hydrophila*** **RK 70363**	97.06	94.58	96.65	95.28	93.69	95.41	87.10	93.56	82.46
64	***Aeromonas*** **sp. 1m**	97.32	94.84	96.90	95.49	93.93	95.41	86.72	93.81	82.71
65	***Aeromonas*** **sp. 12m**	97.19	94.71	96.78	95.41	93.81	95.49	87.10	93.69	82.46
66	***Aeromonas*** **sp. 13m**	98.97	94.71	98.58	95.14	93.81	95.28	86.46	93.93	82.37
67	***Aeromonas*** **sp. 15m**	97.32	94.84	96.90	95.49	93.93	95.62	86.84	93.81	82.58
68	***Aeromonas*** **sp. 17m**	99.74	95.49	99.36	95.88	94.58	96.04	86.72	94.71	83.01
69	***Aeromonas*** **sp. 19m**	97.32	94.84	96.90	95.49	93.93	95.62	87.23	93.81	82.58
70	***A. media*** **CDC 0862-83**	85.44	84.04	85.43	85.06	84.42	85.31	94.46	83.39	80.18
71	***A. culicicola*** **2238A**	83.75	83.01	83.37	82.50	82.24	82.24	80.77	82.58	93.50
72	***A. culicicola*** **3037T**	83.37	82.46	83.14	82.11	81.67	81.67	81.08	82.37	93.76

	10	11	12	13	14	15	16	17	18	19	20	21	22
**1**	36	25	25	46	62	58	31	26	27	37	128	30	133
**2**	5	39	18	35	54	43	39	39	19	26	133	22	129
**3**	39	28	28	49	65	57	34	29	30	40	131	33	136
**4**	23	35	13	28	47	46	33	34	7	18	137	8	135
**5**	8	47	23	40	61	46	45	47	24	31	139	27	137
**6**	26	34	18	33	51	45	36	35	12	23	139	11	140
**7**	106	104	105	102	111	124	106	102	103	109	147	106	147
**8**	53	50	44	57	77	72	49	52	38	46	136	41	144
**9**	139	138	135	137	118	157	140	136	135	140	54	138	117
**10**		39	17	34	55	44	37	39	18	25	133	19	129
**11**	95.02		30	46	65	55	10	13	28	38	135	29	132
**12**	97.81	96.17		25	44	47	26	27	8	19	132	11	132
**13**	95.62	94.12	96.78		31	62	42	43	22	32	132	26	123
**14**	92.90	91.62	94.38	96.04		79	64	62	42	52	122	45	127
**15**	94.38	92.90	93.93	92.08	89.81		59	59	43	54	156	48	152
**16**	95.28	98.71	96.65	94.58	91.75	92.47		13	26	36	130	27	128
**17**	95.02	98.34	96.52	94.46	92.08	92.47	98.34		27	38	130	30	129
**18**	97.69	96.43	98.97	97.19	94.58	94.46	96.65	96.52		13	132	5	131
**19**	96.78	95.14	97.55	95.88	93.36	93.11	95.41	95.14	98.34		132	16	138
**20**	82.71	82.46	83.01	83.01	84.30	79.72	83.27	83.27	83.01	83.01		135	122
**21**	97.55	96.29	98.58	96.65	94.25	93.81	96.52	96.17	99.36	97.94	82.46		131
**22**	83.37	83.14	83.14	84.29	83.62	80.40	83.49	83.37	83.27	82.38	84.30	83.27	
**23**	94.84	98.84	96.29	94.38	91.88	92.65	98.34	98.97	96.29	94.71	83.39	95.88	83.37
**24**	94.84	98.84	96.29	94.38	91.88	92.65	98.34	98.97	96.29	94.71	83.39	95.88	83.37
**25**	96.90	97.19	98.47	96.52	93.93	93.49	97.44	97.06	98.71	97.32	82.58	98.84	83.39
**26**	95.28	98.71	96.65	94.58	92.21	93.11	98.21	98.84	96.65	95.14	83.49	96.29	83.62
**27**	85.56	86.59	85.81	86.46	85.06	83.27	86.84	86.84	86.33	85.43	81.47	86.21	82.59
**28**	95.28	99.74	96.43	94.38	91.88	93.23	98.71	98.34	96.65	95.41	82.71	96.52	83.39
**29**	94.71	98.97	96.17	94.25	91.75	92.52	98.47	98.84	96.17	94.58	83.27	96.04	83.49
**30**	94.38	98.58	95.74	93.81	91.44	92.21	98.08	98.47	95.74	94.25	82.71	95.62	83.27
**31**	95.28	98.71	96.65	94.58	92.21	93.11	98.21	98.84	96.65	95.14	83.49	96.29	83.62
**32**	95.02	98.97	96.43	94.38	91.88	92.77	98.47	99.11	96.43	94.84	83.39	96.04	83.37
**33**	94.38	98.34	95.74	93.81	91.44	92.21	97.81	98.47	95.74	94.25	82.71	95.41	82.71
**34**	94.71	98.71	96.43	94.38	91.88	92.90	98.21	98.84	96.43	95.14	83.37	95.74	83.39
**35**	94.58	98.58	96.29	94.38	91.88	92.65	98.08	98.71	96.29	94.71	83.39	95.62	83.27
**36**	94.71	98.71	96.43	94.46	92.08	92.77	98.21	98.84	96.43	94.84	83.37	95.74	83.39
**37**	94.71	98.71	96.43	94.38	91.88	92.77	98.21	98.84	96.43	94.84	83.37	95.74	83.27
**38**	95.02	98.97	96.43	94.38	91.88	92.90	98.47	99.11	96.43	95.14	83.37	96.04	83.49
**39**	94.84	99.11	96.29	94.25	91.75	92.77	98.58	98.97	96.29	95.02	83.39	96.17	83.62
**40**	94.71	98.97	96.29	94.25	91.75	92.65	98.47	98.84	96.29	94.71	83.27	96.04	83.37
**41**	94.58	98.84	96.17	94.25	91.75	92.52	98.34	98.71	96.17	94.58	83.27	95.88	83.37
**42**	82.58	82.58	82.37	82.37	83.37	79.33	82.71	82.46	82.50	82.50	98.46	82.50	84.41
**43**	94.58	98.84	96.04	94.12	91.62	92.52	98.34	98.97	96.04	94.71	83.14	95.62	83.27
**44**	93.93	97.94	95.62	93.81	91.44	92.08	97.44	98.08	95.62	94.12	82.71	95.02	82.59
**45**	94.84	98.84	96.29	94.38	91.88	92.65	98.34	98.97	96.29	94.71	83.39	95.88	83.37
**46**	97.69	96.65	98.47	96.78	94.12	93.81	96.90	96.29	99.23	98.34	82.71	99.36	83.62
**47**	86.08	86.84	86.33	87.23	86.21	83.37	86.59	86.84	86.46	85.81	80.38	86.46	83.01
**48**	82.37	82.11	82.37	82.37	83.37	79.28	82.50	82.50	82.24	82.24	98.70	81.67	84.17
**49**	97.81	96.04	98.84	97.19	94.46	94.12	96.29	96.17	99.62	98.47	83.14	99.49	83.27
**50**	97.94	96.17	98.71	97.06	94.38	93.93	96.43	96.29	99.49	98.34	83.01	99.62	83.39
**51**	99.49	95.14	97.55	95.41	92.90	94.38	95.14	95.14	97.44	96.52	82.58	97.32	83.49
**52**	94.84	98.84	96.29	94.25	91.88	92.65	98.34	98.71	96.29	94.71	83.27	95.88	83.75
**53**	94.38	98.08	95.74	93.69	91.32	92.21	97.55	98.21	95.74	94.25	83.01	95.41	82.71
**54**	93.69	95.49	94.84	92.21	90.17	90.85	94.71	95.14	94.58	93.36	81.67	94.71	81.56
**55**	99.74	95.02	97.55	95.41	92.65	94.12	95.28	94.71	97.44	96.52	82.71	97.55	83.37
**56**	99.49	94.71	97.32	95.28	92.52	93.81	95.02	94.46	97.19	96.29	82.58	97.06	83.37
**57**	97.69	96.04	98.34	96.65	93.93	93.81	96.29	95.88	99.11	98.08	82.58	99.23	83.27
**58**	94.58	98.84	96.04	93.93	91.49	92.77	98.34	98.71	96.04	94.71	83.14	95.62	83.37
**59**	85.43	86.08	85.69	86.46	85.06	83.01	86.33	86.46	86.21	85.44	81.21	86.08	82.46
**60**	82.59	82.38	82.46	83.75	83.37	80.07	82.71	83.01	82.59	81.69	83.63	82.59	98.21
**61**	83.39	82.59	83.27	84.40	84.04	80.53	83.27	83.39	83.39	82.34	84.41	82.71	98.84
**62**	94.12	97.81	95.49	93.49	91.32	91.88	97.32	97.94	95.49	94.12	82.71	95.14	83.14
**63**	94.71	99.23	96.17	94.12	91.62	92.65	98.71	98.84	96.17	94.84	83.27	96.04	83.75
**64**	94.71	98.47	96.43	94.38	91.88	92.77	97.94	98.58	96.43	94.84	83.37	95.74	83.39
**65**	94.84	98.84	96.29	94.25	91.75	92.65	98.34	98.71	96.29	94.71	83.27	95.88	83.49
**66**	94.84	96.52	96.29	93.36	91.44	91.75	95.74	96.17	95.74	94.46	82.71	95.62	82.71
**67**	95.02	98.71	96.43	94.38	91.88	92.77	98.21	98.84	96.43	94.84	83.39	96.04	83.37
**68**	95.62	97.06	96.78	94.12	92.08	92.52	96.29	96.90	96.52	95.28	83.37	96.43	83.27
**69**	95.02	98.97	96.43	94.38	91.88	92.77	98.47	98.84	96.43	94.84	83.39	96.04	83.62
**70**	84.16	85.31	84.40	85.19	84.16	82.59	84.65	85.31	84.65	84.16	79.33	84.53	80.33
**71**	83.01	83.01	83.14	83.01	84.79	80.05	83.39	83.39	83.14	83.14	98.20	82.58	84.79
**72**	82.46	82.46	82.58	82.46	84.41	79.46	83.01	83.01	82.58	82.58	97.79	82.37	84.41

	23	24	25	26	27	28	29	30	31	32	33	34	35
**1**	22	22	29	21	105	23	23	26	21	21	26	21	22
**2**	40	40	27	38	113	38	41	44	38	39	44	40	40
**3**	25	25	32	24	106	26	26	29	24	24	29	24	25
**4**	36	36	15	33	113	33	37	40	33	35	40	35	36
**5**	48	48	32	45	119	45	49	52	45	47	52	47	48
**6**	35	35	16	32	116	32	36	39	32	34	39	36	37
**7**	103	103	104	102	49	104	104	107	102	102	107	103	105
**8**	49	49	44	48	125	48	50	52	48	48	53	47	48
**9**	134	134	138	132	146	136	135	138	132	134	139	133	134
**10**	40	40	24	37	112	37	41	44	37	39	44	41	42
**11**	9	9	22	10	104	2	8	11	10	8	13	10	11
**12**	29	29	12	26	110	28	30	33	26	28	33	28	29
**13**	44	44	27	42	106	44	45	48	42	44	48	44	44
**14**	63	63	47	61	117	63	64	67	61	63	67	63	63
**15**	57	57	51	54	131	53	58	61	54	56	61	55	57
**16**	13	13	20	14	102	10	12	15	14	12	17	14	15
**17**	8	8	23	9	102	13	9	12	9	7	12	9	10
**18**	29	29	10	26	107	26	30	33	26	28	33	28	29
**19**	41	41	21	38	113	36	42	45	38	40	45	38	41
**20**	129	129	134	127	144	133	130	133	127	129	133	128	129
**21**	32	32	9	29	108	27	31	34	29	31	36	33	34
**22**	129	129	130	127	135	130	128	131	127	129	134	130	131
**23**		0	25	3	105	9	1	4	3	1	6	5	2
**24**	100		25	3	105	9	1	4	3	1	6	5	2
**25**	96.78	96.78		22	105	20	24	27	22	24	29	26	27
**26**	99.62	99.62	97.19		104	8	4	7	0	2	7	4	5
**27**	86.46	86.46	86.46	86.59		104	104	107	104	104	108	105	107
**28**	98.84	98.84	97.44	98.97	86.59		8	11	8	8	13	8	11
**29**	99.87	99.87	96.90	99.49	86.59	98.97		3	4	2	7	6	3
**30**	99.49	99.49	96.52	99.11	86.33	98.58	99.62		7	5	10	9	6
**31**	99.62	99.62	97.19	100	86.59	98.97	99.49	99.11		2	7	4	5
**32**	99.87	99.87	96.90	99.74	86.59	98.97	99.74	99.36	99.74		5	4	3
**33**	99.23	99.23	96.29	99.11	86.21	98.34	99.11	98.71	99.11	99.36		9	8
**34**	99.36	99.36	96.65	99.49	86.46	98.97	99.23	98.84	99.49	99.49	98.84		3
**35**	99.74	99.74	96.52	99.36	86.33	98.58	99.62	99.23	99.36	99.62	98.97	99.62	
**36**	99.87	99.87	96.65	99.49	86.46	98.71	99.74	99.36	99.49	99.74	99.11	99.49	99.87
**37**	99.62	99.62	96.65	99.49	86.46	98.71	99.49	99.11	99.49	99.74	99.36	99.49	99.62
**38**	99.62	99.62	96.90	99.74	86.72	99.23	99.49	99.11	99.74	99.74	99.11	99.74	99.36
**39**	99.49	99.49	97.06	99.62	86.84	99.36	99.62	99.23	99.62	99.62	98.97	99.62	99.23
**40**	99.62	99.62	96.90	99.49	86.59	98.97	99.74	99.36	99.49	99.74	99.11	99.36	99.49
**41**	99.74	99.74	96.78	99.36	86.46	98.84	99.87	99.49	99.36	99.62	98.97	99.23	99.62
**42**	83.01	83.01	82.37	83.27	81.08	83.01	83.14	82.58	83.27	83.01	82.50	83.01	82.71
**43**	99.49	99.49	96.52	99.36	86.46	98.84	99.36	98.97	99.36	99.62	99.49	99.36	99.23
**44**	99.11	99.11	95.88	98.71	85.69	97.94	98.97	98.58	98.71	98.97	98.84	98.71	99.11
**45**	100	100	96.78	99.62	86.46	98.84	99.87	99.49	99.62	99.87	99.23	99.36	99.74
**46**	95.74	95.74	98.97	96.17	86.59	96.90	95.88	95.49	96.17	95.88	95.28	95.88	95.49
**47**	86.72	86.72	86.46	86.84	94.25	86.84	86.84	86.46	86.84	86.84	86.46	86.72	86.46
**48**	82.71	82.71	81.80	83.14	81.08	82.37	82.58	82.37	83.14	82.71	82.37	83.01	82.71
**49**	95.88	95.88	98.58	96.29	86.21	96.29	95.74	95.41	96.29	96.04	95.41	96.04	95.88
**50**	96.04	96.04	98.71	96.43	86.33	96.43	95.88	95.49	96.43	96.17	95.49	95.88	95.74
**51**	95.02	95.02	96.65	95.28	85.56	95.28	94.84	94.46	95.28	95.14	94.46	94.71	94.71
**52**	99.49	99.49	97.06	99.62	86.72	99.11	99.36	98.97	99.62	99.62	98.97	99.36	99.23
**53**	98.97	98.97	96.29	99.11	86.08	98.34	98.84	98.47	99.11	99.11	98.47	98.84	98.71
**54**	95.62	95.62	94.84	95.74	85.31	95.74	95.74	95.41	95.74	95.74	95.14	95.62	95.49
**55**	94.58	94.58	96.90	95.02	85.81	95.28	94.71	94.38	95.02	94.71	94.12	94.46	94.38
**56**	94.38	94.38	96.65	94.71	85.44	95.02	94.25	93.81	94.71	94.46	93.81	94.25	94.12
**57**	95.88	95.88	98.34	96.29	85.81	96.29	95.74	95.41	96.29	96.04	95.41	95.74	95.62
**58**	99.23	99.23	96.78	99.36	86.72	99.11	99.11	98.71	99.36	99.36	98.71	99.36	98.97
**59**	86.21	86.21	86.21	86.33	98.71	86.08	86.33	85.81	86.33	86.33	85.69	86.21	85.81
**60**	83.01	83.01	82.46	83.27	82.38	82.46	83.14	82.59	83.27	83.01	82.38	82.71	82.59
**61**	83.39	83.39	83.01	83.49	82.59	83.01	83.27	82.71	83.49	83.39	82.59	83.37	83.39
**62**	98.71	98.71	96.04	98.84	86.08	98.08	98.58	98.21	98.84	98.84	98.21	98.58	98.47
**63**	99.36	99.36	97.19	99.49	87.10	99.49	99.49	99.11	99.49	99.49	98.84	99.49	99.11
**64**	99.36	99.36	96.65	99.49	86.46	98.71	99.23	98.84	99.49	99.49	98.84	99.49	99.36
**65**	99.49	99.49	97.06	99.62	86.72	99.11	99.36	98.97	99.62	99.62	98.97	99.36	99.23
**66**	96.65	96.65	96.04	96.78	86.46	96.78	96.52	96.17	96.78	96.78	96.17	96.52	96.43
**67**	99.62	99.62	96.90	99.74	86.59	98.97	99.49	99.11	99.74	99.74	99.11	99.49	99.36
**68**	97.44	97.44	96.52	97.55	86.72	97.32	97.32	96.90	97.55	97.55	96.90	97.32	97.19
**69**	99.62	99.62	97.19	99.74	86.84	99.23	99.49	99.11	99.74	99.74	99.11	99.49	99.36
**70**	85.44	85.44	84.53	85.43	92.47	85.31	85.43	85.19	85.43	85.43	85.06	85.44	85.19
**71**	83.63	83.63	82.71	84.04	81.34	83.27	83.49	83.27	84.04	83.63	83.27	83.75	83.63
**72**	83.39	83.39	82.50	83.49	81.47	82.71	83.27	82.71	83.49	83.39	82.71	83.37	83.39

	36	37	38	39	40	41	42	43	44	45	46	47	48
**1**	21	21	21	22	22	23	130	24	27	22	31	104	132
**2**	39	39	40	41	40	41	136	42	45	40	21	110	138
**3**	24	24	24	25	25	26	133	27	30	25	34	105	135
**4**	35	35	35	36	36	37	141	38	41	36	11	111	143
**5**	47	47	47	48	48	49	141	50	53	48	26	116	143
**6**	36	36	34	35	36	37	142	37	42	35	14	114	145
**7**	104	104	101	102	104	105	151	104	110	103	102	38	149
**8**	48	48	48	49	49	50	138	51	54	49	42	122	140
**9**	133	134	133	134	135	135	59	136	139	134	136	150	51
**10**	41	41	39	40	41	42	134	42	47	40	18	109	137
**11**	10	10	8	7	8	9	134	9	16	9	26	102	139
**12**	28	28	28	29	29	30	137	31	34	29	12	107	137
**13**	43	44	44	45	45	45	137	46	48	44	25	100	137
**14**	62	63	63	64	64	64	128	65	67	63	46	108	128
**15**	56	56	55	56	57	58	159	58	62	57	48	129	161
**16**	14	14	12	11	12	13	133	13	20	13	24	104	136
**17**	9	9	7	8	9	10	135	8	15	8	29	102	136
**18**	28	28	28	29	29	30	136	31	34	29	6	105	138
**19**	40	40	38	39	41	42	136	41	46	41	13	110	138
**20**	128	128	128	129	130	130	12	131	133	129	133	151	10
**21**	33	33	31	30	31	32	136	34	39	32	5	106	141
**22**	130	131	128	127	129	129	120	131	135	129	127	133	123
**23**	1	3	3	4	3	2	132	4	7	0	33	103	133
**24**	1	3	3	4	3	2	132	4	7	0	33	103	133
**25**	26	26	24	23	24	25	137	27	32	25	8	105	140
**26**	4	4	2	3	4	5	130	5	10	3	30	102	131
**27**	106	106	103	102	104	105	147	105	111	105	104	45	147
**28**	10	10	6	5	8	9	132	9	16	9	24	102	137
**29**	2	4	4	3	2	1	131	5	8	1	32	102	134
**30**	5	7	7	6	5	4	134	8	11	4	35	105	137
**31**	4	4	2	3	4	5	130	5	10	3	30	102	131
**32**	2	2	2	3	2	3	132	3	8	1	32	102	133
**33**	7	5	7	8	7	8	136	4	9	6	37	106	137
**34**	4	4	2	3	5	6	132	5	10	5	32	103	132
**35**	1	3	5	6	4	3	133	6	7	2	35	105	133
**36**		2	4	5	3	2	132	5	6	1	34	104	132
**37**	99.74		4	5	3	4	132	5	8	3	34	104	132
**38**	99.49	99.49		1	4	5	131	3	10	3	30	101	132
**39**	99.36	99.36	99.87		3	4	130	4	11	4	29	100	133
**40**	99.62	99.62	99.49	99.62		1	130	5	9	3	32	102	134
**41**	99.74	99.49	99.36	99.49	99.87		130	6	8	2	33	103	134
**42**	83.01	83.01	83.14	83.27	83.27	83.27		134	137	132	134	149	8
**43**	99.36	99.36	99.62	99.49	99.36	99.23	82.58		7	4	33	103	135
**44**	99.23	98.97	98.71	98.58	98.84	98.97	82.37	99.11		7	40	109	137
**45**	99.87	99.62	99.62	99.49	99.62	99.74	83.01	99.49	99.11		33	103	133
**46**	95.62	95.62	96.17	96.29	95.88	95.74	82.58	95.74	94.84	95.74		102	139
**47**	86.59	86.59	87.10	87.23	86.84	86.72	80.63	86.72	86.08	86.72	86.84		149
**48**	83.01	83.01	83.01	82.71	82.58	82.58	98.96	82.46	82.37	82.71	82.11	80.63	
**49**	96.04	96.04	96.04	95.88	95.88	95.74	82.46	95.62	95.28	95.88	99.36	86.46	82.37
**50**	95.88	95.88	96.17	96.04	95.88	95.74	82.46	95.74	95.14	96.04	99.49	86.46	82.24
**51**	94.84	94.84	95.02	94.84	94.84	94.71	82.50	94.71	94.12	95.02	97.44	86.08	82.11
**52**	99.36	99.36	99.62	99.49	99.36	99.23	82.71	99.23	98.58	99.49	96.04	87.35	82.58
**53**	98.84	98.84	99.11	98.97	98.84	98.71	82.46	98.71	98.08	98.97	95.28	86.33	82.50
**54**	95.49	95.49	95.74	95.88	95.74	95.62	81.80	95.41	94.71	95.62	94.58	85.56	81.34
**55**	94.46	94.46	94.71	94.84	94.71	94.58	83.01	94.38	93.69	94.58	97.69	86.33	82.37
**56**	94.25	94.25	94.46	94.38	94.25	94.12	82.46	94.12	93.49	94.38	97.44	85.81	82.24
**57**	95.74	95.74	96.04	95.88	95.74	95.62	82.37	95.62	95.02	95.88	99.11	86.21	81.80
**58**	99.11	99.11	99.62	99.49	99.11	98.97	82.58	99.23	98.34	99.23	96.04	87.10	82.46
**59**	86.08	86.08	86.46	86.46	86.33	86.21	80.63	86.21	85.44	86.21	86.33	93.69	80.63
**60**	82.71	82.59	83.14	83.27	83.01	83.01	84.04	82.59	82.25	83.01	83.01	82.46	83.49
**61**	83.37	83.39	83.37	83.39	83.27	83.27	84.42	83.14	82.71	83.39	83.39	83.01	84.42
**62**	98.58	98.58	98.84	98.71	98.58	98.47	82.50	98.47	97.81	98.71	95.02	86.33	82.37
**63**	99.23	99.23	99.74	99.87	99.49	99.36	83.14	99.36	98.47	99.36	96.43	87.35	82.58
**64**	99.49	99.49	99.49	99.36	99.36	99.23	83.01	99.11	98.71	99.36	95.62	86.72	83.01
**65**	99.36	99.36	99.62	99.49	99.36	99.23	82.71	99.23	98.58	99.49	96.04	87.10	82.58
**66**	96.52	96.52	96.78	96.65	96.52	96.43	82.58	96.43	95.74	96.65	95.74	86.46	82.37
**67**	99.49	99.49	99.74	99.62	99.49	99.36	83.01	99.36	98.71	99.62	95.88	86.84	82.71
**68**	97.32	97.32	97.55	97.44	97.32	97.19	83.39	97.19	96.52	97.44	96.29	86.84	83.01
**69**	99.49	99.49	99.74	99.62	99.49	99.36	83.01	99.36	98.71	99.62	96.17	87.23	82.71
**70**	85.31	85.31	85.56	85.69	85.43	85.44	79.28	85.44	84.53	85.44	84.78	96.52	79.33
**71**	83.75	83.75	83.75	83.63	83.49	83.49	97.16	83.37	83.27	83.63	83.01	80.63	97.43
**72**	83.37	83.37	83.37	83.39	83.27	83.27	96.75	83.14	82.71	83.39	82.46	80.77	97.43

	49	50	51	52	53	54	55	56	57	58	59	60	61
**1**	28	29	36	22	26	19	38	40	30	24	108	137	132
**2**	18	19	1	41	45	50	7	9	21	43	114	135	128
**3**	31	32	39	25	29	22	41	43	33	25	107	138	135
**4**	6	7	25	36	40	47	25	27	10	38	110	140	135
**5**	23	24	12	48	52	55	10	12	26	48	115	143	136
**6**	11	10	28	35	39	44	28	30	13	37	112	144	141
**7**	104	103	106	101	105	113	106	108	106	101	55	150	145
**8**	39	40	55	49	53	54	55	56	42	49	126	146	143
**9**	134	135	138	134	137	144	139	141	138	136	145	122	118
**10**	17	16	4	40	44	49	2	4	18	42	113	135	130
**11**	31	30	38	9	15	35	39	41	31	9	109	138	135
**12**	9	10	19	29	33	40	19	21	13	31	111	136	131
**13**	22	23	36	45	49	61	36	37	26	47	106	126	121
**14**	43	44	55	63	68	77	57	58	47	66	117	129	125
**15**	46	47	44	57	61	71	46	48	48	56	133	156	151
**16**	29	28	38	13	19	41	37	39	29	13	107	134	131
**17**	30	29	38	10	14	38	41	43	32	10	105	133	130
**18**	3	4	20	29	33	42	20	22	7	31	108	135	130
**19**	12	13	27	41	45	52	27	29	15	41	114	142	137
**20**	131	132	134	130	132	141	133	134	134	131	146	126	120
**21**	4	3	21	32	36	41	19	23	6	34	109	135	134
**22**	131	130	128	126	134	143	129	129	131	129	136	14	9
**23**	32	31	39	4	8	34	42	44	32	6	108	133	130
**24**	32	31	39	4	8	34	42	44	32	6	108	133	130
**25**	11	10	26	23	29	40	24	26	13	25	108	136	133
**26**	29	28	37	3	7	33	39	41	29	5	107	131	128
**27**	108	107	112	103	109	115	110	114	110	103	10	138	135
**28**	29	28	37	7	13	33	37	39	29	7	109	136	133
**29**	33	32	40	5	9	33	41	45	33	7	107	132	131
**30**	36	35	43	8	12	36	44	48	36	10	110	135	134
**31**	29	28	37	3	7	33	39	41	29	5	107	131	128
**32**	31	30	38	3	7	33	41	43	31	5	107	133	130
**33**	36	35	43	8	12	38	46	48	36	10	111	138	135
**34**	31	32	41	5	9	34	43	45	33	5	108	134	129
**35**	32	33	41	6	10	35	44	46	34	8	110	135	130
**36**	31	32	40	5	9	35	43	45	33	7	109	134	129
**37**	31	32	40	5	9	35	43	45	33	7	109	135	130
**38**	31	30	39	3	7	33	41	43	31	3	106	132	129
**39**	32	31	40	4	8	32	40	44	32	4	105	131	130
**40**	32	32	40	5	9	33	41	45	33	7	107	133	131
**41**	33	33	41	6	10	34	42	46	34	8	108	133	131
**42**	135	135	136	133	135	140	132	135	137	134	149	124	121
**43**	34	33	41	6	10	36	44	46	34	6	108	135	132
**44**	37	38	46	11	15	41	49	51	39	13	114	139	134
**45**	32	31	39	4	8	34	42	44	32	6	108	133	130
**46**	5	4	20	31	37	42	18	20	7	31	107	133	130
**47**	106	105	109	99	107	112	107	110	108	101	49	136	133
**48**	137	138	139	134	136	145	137	138	140	135	149	127	121
**49**		1	19	32	36	43	19	21	4	34	109	135	130
**50**	99.87		18	31	35	42	18	20	3	33	108	134	131
**51**	97.55	97.69		40	44	49	6	8	20	42	113	134	129
**52**	95.88	96.04	94.84		8	34	42	41	32	4	108	132	127
**53**	95.41	95.49	94.38	98.97		38	46	48	36	10	112	137	135
**54**	94.46	94.58	93.69	95.62	95.14		49	53	42	36	117	147	146
**55**	97.55	97.69	99.23	94.58	94.12	93.69		6	20	44	111	135	132
**56**	97.32	97.44	98.97	94.71	93.81	93.23	99.23		22	44	117	137	130
**57**	99.49	99.62	97.44	95.88	95.41	94.58	97.44	97.19		34	111	135	132
**58**	95.62	95.74	94.58	99.49	98.71	95.41	94.38	94.38	95.62		108	133	130
**59**	86.08	86.21	85.43	86.21	85.56	85.06	85.69	85.06	85.69	86.21		137	136
**60**	82.59	82.71	82.71	83.14	82.34	81.22	82.59	82.34	82.59	83.01	82.34		17
**61**	83.39	83.27	83.37	83.62	82.59	81.35	83.14	83.39	83.14	83.39	82.46	97.81	
**62**	95.14	95.28	94.12	98.71	98.21	95.41	93.81	93.56	95.14	98.47	85.56	82.34	82.71
**63**	95.74	95.88	94.71	99.62	98.84	95.74	94.71	94.46	95.74	99.62	86.46	83.14	83.37
**64**	96.04	95.88	94.71	99.36	98.84	95.74	94.46	94.25	95.74	99.11	86.21	82.71	83.37
**65**	95.88	96.04	94.84	99.74	98.97	95.88	94.58	94.58	96.04	99.49	86.21	82.71	83.37
**66**	95.62	95.74	94.84	96.90	96.17	97.06	94.58	94.58	95.62	96.65	85.69	82.12	82.59
**67**	96.04	96.17	95.02	99.62	99.11	95.74	94.71	94.46	96.04	99.36	86.33	83.01	83.39
**68**	96.43	96.52	95.62	97.44	96.90	97.81	95.41	95.14	96.43	97.19	86.46	82.59	83.14
**69**	96.04	96.17	95.02	99.87	99.11	95.74	94.71	94.71	96.04	99.62	86.33	83.01	83.49
**70**	84.53	84.65	84.16	85.44	85.19	84.29	84.42	83.49	84.42	85.44	92.77	80.21	80.33
**71**	83.27	83.14	82.71	83.63	83.39	82.24	82.58	82.58	82.71	83.37	81.08	84.17	85.20
**72**	82.71	82.58	82.50	83.39	83.01	81.67	82.37	82.37	82.50	83.14	81.21	83.63	84.66

	62	63	64	65	66	67	68	69	70	71	72
**1**	28	23	21	22	8	21	2	21	114	125	128
**2**	47	42	40	41	41	40	35	40	125	132	135
**3**	31	26	24	25	11	24	5	24	113	128	131
**4**	40	37	35	36	38	35	32	35	117	136	139
**5**	54	49	47	48	48	47	42	47	122	138	141
**6**	41	36	36	35	37	34	31	34	115	138	141
**7**	107	101	103	101	106	102	103	100	43	148	147
**8**	55	50	48	49	47	48	41	48	130	134	137
**9**	136	135	133	135	137	134	132	134	154	50	48
**10**	46	41	41	40	40	39	34	39	124	132	135
**11**	17	6	12	9	27	10	23	8	115	132	135
**12**	35	30	28	29	29	28	25	28	121	131	134
**13**	51	46	44	45	52	44	46	44	116	132	135
**14**	68	65	63	64	67	63	62	63	124	117	120
**15**	63	57	56	57	64	56	58	56	135	155	158
**16**	21	10	16	13	33	14	29	12	119	129	132
**17**	16	9	11	10	30	9	24	9	115	129	132
**18**	35	30	28	29	33	28	27	28	119	131	134
**19**	46	40	40	41	43	40	37	40	124	131	134
**20**	133	130	128	130	133	129	128	129	159	14	17
**21**	38	31	33	32	34	31	28	31	120	134	137
**22**	132	126	130	128	134	129	131	127	154	117	120
**23**	10	5	5	4	26	3	20	3	114	126	129
**24**	10	5	5	4	26	3	20	3	114	126	129
**25**	31	22	26	23	31	24	27	22	120	133	136
**26**	9	4	4	3	25	2	19	2	113	124	127
**27**	109	101	105	103	106	104	103	102	59	145	144
**28**	15	4	10	7	25	8	21	6	115	130	133
**29**	11	4	6	5	27	4	21	4	113	127	130
**30**	14	7	9	8	30	7	24	7	116	130	133
**31**	9	4	4	3	25	2	19	2	113	124	127
**32**	9	4	4	3	25	2	19	2	113	126	129
**33**	14	9	9	8	30	7	24	7	117	130	133
**34**	11	4	4	5	27	4	21	4	114	125	128
**35**	12	7	5	6	28	5	22	5	116	126	129
**36**	11	6	4	5	27	4	21	4	115	125	128
**37**	11	6	4	5	27	4	21	4	115	125	128
**38**	9	2	4	3	25	2	19	2	112	125	128
**39**	10	1	5	4	26	3	20	3	111	126	129
**40**	11	4	5	5	27	4	21	4	113	127	130
**41**	12	5	6	6	28	5	22	5	114	127	130
**42**	136	131	132	133	134	132	129	132	161	22	25
**43**	12	5	7	6	28	5	22	5	114	128	131
**44**	17	12	10	11	33	10	27	10	120	130	133
**45**	10	5	5	4	26	3	20	3	114	126	129
**46**	39	28	34	31	33	32	29	30	118	132	135
**47**	107	99	103	101	105	102	102	100	27	149	148
**48**	137	134	132	134	137	133	132	133	159	20	20
**49**	38	33	31	32	34	31	28	31	120	130	133
**50**	37	32	32	31	33	30	27	30	119	131	134
**51**	46	41	41	40	40	39	34	39	124	133	136
**52**	10	3	5	2	24	3	20	1	114	126	129
**53**	14	9	9	8	30	7	24	7	116	129	132
**54**	36	33	33	32	23	33	17	33	123	138	141
**55**	48	41	43	42	42	41	36	41	122	134	137
**56**	50	43	45	42	42	43	38	41	128	134	137
**57**	38	33	33	31	34	31	28	31	122	133	136
**58**	12	3	7	4	26	5	22	3	114	128	131
**59**	112	106	108	108	111	107	106	107	56	147	146
**60**	137	132	134	134	140	133	135	133	155	123	126
**61**	134	129	129	129	135	130	132	128	154	115	118
**62**		11	9	10	32	9	26	9	118	130	133
**63**	98.58		6	3	25	4	21	2	112	127	130
**64**	98.84	99.23		5	27	4	21	4	114	125	128
**65**	98.71	99.62	99.36		24	3	20	1	114	127	130
**66**	95.88	96.78	96.52	96.90		25	6	23	119	130	133
**67**	98.84	99.49	99.49	99.62	96.78		19	2	113	126	129
**68**	96.65	97.32	97.32	97.44	99.23	97.55		19	114	125	128
**69**	98.84	99.74	99.49	99.87	97.06	99.74	97.55		113	126	129
**70**	84.78	85.56	85.44	85.44	84.65	85.43	85.44	85.43		158	157
**71**	83.27	83.49	83.75	83.49	83.27	83.63	83.75	83.63	79.46		3
**72**	82.71	83.27	83.37	83.27	82.71	83.39	83.37	83.39	79.60	99.61	
