# Supplementary material for: luxRI homologs are universally present in the genus Aeromonas
Source: BMC Microbiol. 2007 Oct 23;7:93. doi: 10.1186/1471-2180-7-93 (PMC2180181; doi:10.1186/1471-2180-7-93)
Supplement: Additional file 2 — Similarity Matrix for the luxI homologs. Percentage sequence similarity (lower left) and number of nucleotide differences (top right). [file 1471-2180-7-93-S2.pdf]

Additional Table 2: Similarity Matrix for the *luxI* homologs. Percentage sequence similarity (lower left) and number of nucleotide differences (top right).

		1	2	3	4	5	6	7	8	9	10	11	12	13	14	15	16	17	18	19
**1**	***A. hydrophila*** **(X89469)**		74	13	47	74	178	69	184	148	41	63	41	33	35	46	39	181	40	181
**2**	***A. salmonicida*** **(U65741)**	88.14		70	53	13	176	44	179	147	67	77	72	60	62	77	64	179	59	178
**3**	***A. hydrophila*** **ATCC 7966T**	97.90	88.67		43	70	179	66	182	143	42	60	40	34	36	47	40	179	35	179
**4**	***A. bestiarum*** **ATCC 51108T**	92.47	91.43	93.11		54	184	43	187	147	55	67	57	52	54	63	58	183	46	184
**5**	***A. hydrophila*** **CDC 0434-84**	88.14	97.90	88.67	91.34		171	44	176	144	66	78	70	59	61	76	63	178	58	177
**6**	***A. sobria*** **CIP 7433T**	71.43	71.47	71.27	70.47	72.29		179	61	99	177	182	178	172	173	185	171	64	176	60
**7**	***A. popoffii*** **LMG 17541T**	88.83	92.87	89.42	93.11	92.87	71.27		181	152	71	82	73	65	67	80	69	181	60	180
**8**	***A. culicicola*** **MTCC 3249T**	70.47	71.27	70.50	69.68	71.47	90.45	70.66		129	180	186	184	177	178	191	176	10	180	6
**9**	***A. veronii*** **bt** ***sobria*** **AE-21**	76.24	76.41	77.05	76.41	76.66	84.36	75.36	79.61		144	153	145	146	147	154	149	130	143	127
**10**	***Aeromonas*** **sp. AE-51**	93.42	89.24	93.26	91.17	89.41	71.27	88.49	71.06	76.62		35	20	19	17	26	19	178	25	178
**11**	***A. hydrophila*** **AE-53**	89.76	87.48	90.34	89.21	87.44	70.36	86.67	70	75.32	94.35		43	36	36	40	38	183	39	183
**12**	***A. hydrophila*** **AE-57**	93.43	88.46	93.53	90.78	88.67	71.43	88.30	70.47	76.49	96.76	93.07		26	24	17	28	181	30	181
**13**	***A. hydrophila*** **AN-1**	94.67	90.38	94.50	91.59	90.45	72.39	89.49	71.31	76.33	96.92	94.21	95.79		2	29	6	174	20	174
**14**	***A. hydrophila*** **AN-2**	94.39	90.06	94.23	91.34	90.23	72.23	89.26	71.43	76.41	97.27	94.21	96.15	99.68		27	4	175	22	175
**15**	***Aeromonas*** **sp. AN-24**	92.56	87.54	92.47	89.81	87.70	70.30	87.18	69.34	75.28	95.79	93.50	97.27	95.35	95.63		31	188	35	188
**16**	***A. hydrophila*** **AN-32**	93.69	89.64	93.53	90.62	89.81	72.29	88.83	71.47	76.08	96.92	93.82	95.47	99.04	99.36	95.03		173	26	173
**17**	***A. trota*** **AN-35**	70.66	71.27	71.27	70.33	71.43	89.88	70.66	98.44	79.46	71.38	70.48	70.66	72.07	71.63	69.52	72.23		177	4
**18**	***Aeromonas*** **sp. AN-46**	93.53	90.45	94.39	92.56	90.62	71.47	90.38	71.11	77.05	95.95	93.66	95.19	96.76	96.48	94.39	95.79	71.31		177
**19**	***A. veronii*** **CECT 4246**	70.66	71.43	71.27	70.47	71.31	90.52	71.11	99.07	80.13	71.38	70.48	70.66	72.07	71.63	69.52	72.23	99.37	71.31	
**20**	***A. bestiarum*** **LMG 13448**	70.66	71.43	71.27	70.47	71.31	90.52	71.11	99.07	80.13	71.38	70.48	70.66	72.07	71.63	69.52	72.23	99.37	71.31	100
**21**	***A. bestiarum*** **LMG 13662**	71.11	71.31	71.43	70.33	71.47	90.45	71.27	98.90	79.77	71.27	70.36	71.11	72.23	72.07	69.68	72.39	99.53	71.47	99.84
**22**	***A. salmonicida*** **CECT 894T**	88.51	99.52	89.26	91.91	98.08	71.63	93.43	71.43	76.33	89.63	87.64	88.83	90.78	90.45	88.14	90.23	71.43	91.02	71.31
**23**	***Aeromonas*** **sp. Manipal A1**	94.39	90.45	94.23	91.59	90.62	72.44	89.26	71.47	76.49	97.27	94.47	96.48	98.71	98.40	96.31	97.73	72.23	96.48	72.23
**24**	***Aeromonas*** **sp. BJMC**	94.39	90.62	94.50	91.91	90.78	72.23	89.64	71.27	76.49	97.43	94.36	96.61	98.54	98.54	95.79	97.90	71.47	96.92	71.47
**25**	***A. hydrophila*** **ATCC 49140**	98.24	88.46	98.40	92.72	88.67	70.33	89.42	70.15	76.08	93.26	90.02	93.53	94.82	94.50	92.72	93.85	70.47	94.07	70.47
**26**	***A. bestiarum*** **ATCC 13444**	88.51	97.43	88.83	91.43	99.19	72.23	93.11	71.47	76.33	89.47	87.48	88.83	90.62	90.38	87.86	90.06	71.43	90.78	71.31
**27**	***A. bestiarum*** **ATCC 23211**	88.46	96.61	88.67	91.02	98.08	72.07	92.72	71.31	76.49	89.47	87.48	88.83	90.45	90.23	87.86	89.81	71.27	90.78	71.43
**28**	***A. bestiarum*** **ATCC 23213**	92.30	91.91	92.47	97.27	91.75	70.66	94.07	70.33	76.33	90.60	88.24	90.23	91.18	90.78	89.42	90.23	70.33	91.91	70.50
**29**	***A. hydrophila*** **RK 217215**	93.85	90.06	93.69	91.34	89.81	72.07	89.42	71.43	76.66	96.47	95.17	95.47	97.90	97.58	95.03	96.92	71.63	96.31	71.63
**30**	***Aeromonas*** **sp. 1m**	95.35	90.62	94.82	92.47	90.45	71.63	90.23	71.11	77.37	96.60	93.50	96.15	98.08	98.08	95.63	97.43	71.31	97.43	71.31
**31**	***Aeromonas*** **sp. 12m**	95.19	90.45	94.67	92.30	90.38	71.31	90.06	70.50	77.05	96.15	93.07	95.63	97.90	97.58	95.19	96.92	71.27	97.27	71.27
**32**	***Aeromonas*** **sp. 13m**	97.43	88.51	97.90	92.47	88.51	70.30	88.83	69.52	76.41	92.55	90.02	93.27	93.85	93.53	92.15	92.87	70.30	93.53	70.30
**33**	***Aeromonas*** **sp. 15m**	95.47	90.78	95.03	92.56	90.62	71.63	90.38	71.11	77.37	96.47	93.40	95.95	98.24	97.90	95.47	97.27	71.31	97.58	71.31
**34**	***Aeromonas*** **sp. 19m**	95.03	90.38	94.50	92.15	90.23	71.63	89.81	71.11	77.37	96.30	93.50	95.47	97.73	97.43	95.35	97.12	71.31	97.12	71.31
**35**	***A. culicicola*** **2238A**	70.66	71.43	71.27	70.47	71.31	90.52	71.11	98.44	80.44	71.38	70	70.66	71.63	71.47	69.52	72.07	98.12	71.43	98.74
**36**	***A. culicicola*** **3037T**	70.50	71.27	71.11	70.30	71.43	90.30	70.66	98.12	80.13	71.22	69.54	70.50	71.47	71.31	69.36	71.63	97.79	71.27	98.44

		20	21	22	23	24	25	26	27	28	29	30	31	32	33	34	35	36
1	***A. hydrophila* (X89469)**	181	180	71	35	35	11	71	72	48	38	29	30	16	28	31	181	182
2	***A. salmonicida* (U65741)**	178	177	3	59	58	72	16	21	50	62	58	59	71	57	60	178	179
3	***A. hydrophila* ATCC 7966^{T}**	179	178	67	36	34	10	69	70	47	39	32	33	13	31	34	179	180
4	***A. bestiarum* ATCC 51108^{T}**	184	183	50	52	50	45	53	56	17	54	47	48	47	46	49	184	185
5	***A. hydrophila* CDC 0434-84**	177	176	12	58	57	70	5	12	51	63	59	60	71	58	61	177	178
6	***A. sobria* CIP 7433^{T}**	60	61	175	170	173	183	173	174	181	174	175	177	185	175	175	60	62
7	***A. popoffii* LMG 17541^{T}**	180	179	41	67	64	66	43	45	37	66	61	62	69	60	63	180	181
8	***A. culicicola* MTCC 3249^{T}**	6	7	178	176	179	186	176	177	183	178	180	182	188	180	180	10	12
9	***A. veronii* bt *sobria* AE-21**	127	128	146	145	145	149	146	145	146	144	141	143	147	141	141	125	127
10	***Aeromonas* sp. AE-51**	178	177	64	17	16	42	65	65	58	22	21	24	46	22	23	178	179
11	***A. hydrophila* AE-53**	183	182	76	34	35	62	77	77	73	30	40	43	62	41	40	186	187
12	***A. hydrophila* AE-57**	181	180	69	22	21	40	69	69	61	28	24	27	42	25	28	181	182
13	***A. hydrophila* AN-1**	174	173	57	8	9	32	58	59	55	13	12	13	38	11	14	175	176
14	***A. hydrophila* AN-2**	175	174	59	10	9	34	60	61	57	15	12	15	40	13	16	176	177
15	***Aeromonas* sp. AN-24**	188	187	74	23	26	45	75	75	66	31	27	30	49	28	29	188	189
16	***A. hydrophila* AN-32**	173	172	61	14	13	38	62	63	61	19	16	19	44	17	18	174	175
17	***A. trota* AN-35**	4	3	178	173	176	184	178	179	183	175	177	179	185	177	177	12	14
18	***Aeromonas* sp. AN-46**	177	176	56	22	19	37	57	57	50	23	16	17	40	15	18	178	179
19	***A. veronii* CECT 4246**	0	1	177	173	176	184	177	178	182	175	177	179	185	177	177	8	10
20	***A. bestiarum* LMG 13448**		1	177	173	176	184	177	178	182	175	177	179	185	177	177	8	10
21	***A. bestiarum* LMG 13662**	99.84		176	172	175	183	176	177	181	174	176	178	184	176	176	9	11
22	***A. salmonicida* CECT 894^{T}**	71.31	71.47		56	55	69	13	18	47	61	55	56	68	54	57	177	178
23	***Aeromonas* sp. Manipal A1**	72.23	72.39	91.02		7	34	57	59	55	13	14	15	38	13	16	173	174
24	***Aeromonas* sp. BJMC**	71.47	71.63	91.18	98.87		34	56	56	53	16	13	16	36	14	17	176	177
25	***A. hydrophila* ATCC 49140**	70.47	70.33	88.83	94.50	94.50		67	68	45	38	28	29	12	27	30	184	185
26	***A. bestiarum* ATCC 13444**	71.31	71.47	97.90	90.78	91.02	89.26		7	48	60	56	57	68	55	58	177	178
27	***A. bestiarum* ATCC 23211**	71.43	71.31	97.12	90.45	91.02	89.10	98.87		51	60	57	58	69	56	59	178	179
28	***A. bestiarum* ATCC 23213**	70.50	70.66	92.47	91.18	91.43	92.72	92.30	91.75		55	48	49	48	47	50	182	183
29	***A. hydrophila* RK 217215**	71.63	72.07	90.23	97.90	97.43	93.85	90.38	90.38	91.18		19	20	42	18	21	176	177
30	***Aeromonas* sp. 1m**	71.31	71.47	91.18	97.73	97.90	95.47	91.02	90.78	92.30	96.92		3	33	1	4	177	178
31	***Aeromonas* sp. 12m**	71.27	71.43	91.02	97.58	97.43	95.35	90.78	90.62	92.15	96.76	99.52		34	2	5	179	180
32	***Aeromonas* sp. 13m**	70.30	70.47	89.10	93.85	94.23	98.08	89.10	88.83	92.30	93.27	94.67	94.50		32	35	185	186
33	***Aeromonas* sp. 15m**	71.31	71.47	91.34	97.90	97.73	95.63	91.18	91.02	92.47	97.12	99.84	99.68	94.82		3	177	178
34	***Aeromonas* sp. 19m**	71.31	71.47	90.78	97.43	97.27	95.19	90.62	90.45	91.91	96.61	99.36	99.19	94.39	99.52		177	178
35	***A. culicicola* 2238A**	98.74	98.58	71.31	72.23	71.47	70.47	71.31	71.43	70.50	71.47	71.31	71.27	70.30	71.31	71.31		2
36	***A. culicicola* 3037T**	98.44	98.28	71.43	72.07	71.31	70.30	71.43	71.27	70.33	71.31	71.43	71.11	70.15	71.43	71.43	99.69	
